# Enhanced convolutional neural network for plankton identification and enumeration

**DOI:** 10.1371/journal.pone.0219570

**Published:** 2019-07-10

**Authors:** Kaichang Cheng, Xuemin Cheng, Yuqi Wang, Hongsheng Bi, Mark C. Benfield

**Affiliations:** 1 Graduate School at Shenzhen, Tsinghua University, Shenzhen, Guangdong, P.R. China; 2 Chesapeake Biological Laboratory, University of Maryland Center for Environmental Science, Solomons, Maryland, United States of America; 3 Department of Oceanography and Coastal Sciences, Louisiana State University, Baton Rouge, Louisiana, United States of America; Newcastle University, UNITED KINGDOM

## Abstract

Despite the rapid increase in the number and applications of plankton imaging systems in marine science, processing large numbers of images remains a major challenge due to large variations in image content and quality in different marine environments. We constructed an automatic plankton image recognition and enumeration system using an enhanced Convolutional Neural Network (CNN) and examined the performance of different network structures on automatic plankton image classification. The procedure started with an adaptive thresholding approach to extract Region of Interest (ROIs) from *in situ* plankton images, followed by a procedure to suppress the background noise and enhance target features for each extracted ROI. The enhanced ROIs were classified into seven categories by a pre-trained classifier which was a combination of a CNN and a Support Vector Machine (SVM). The CNN was selected to improve feature description and the SVM was utilized to improve classification accuracy. A series of comparison experiments were then conducted to test the effectiveness of the pre-trained classifier including the combination of CNN and SVM versus CNN alone, and the performance of different CNN models. Compared to CNN model alone, the combination of CNN and SVM increased classification accuracy and recall rate by 7.13% and 6.41%, respectively. Among the selected CNN models, the ResNet50 performed the best with accuracy and recall at 94.52% and 94.13% respectively. The present study demonstrates that deep learning technique can improve plankton image recognition and that the results can provide useful information on the selection of different CNN models for plankton recognition. The proposed algorithm could be generally applied to images acquired from different imaging systems.

## Introduction

Zooplankton play a pivotal role in marine ecosystems by feeding on phytoplankton and serving as important food for fish larvae [1]. Understanding their spatial and temporal dynamics and interactions with their environment remain fundamental questions in plankton ecology [2]. In recent years, imaging techniques have contributed greatly to our understanding of fine-scale plankton distributions and their interactions with their environments [3]. The number of imaging systems and applications have greatly increased in recent years, e.g., Video Plankton Recorder(VPR) [4], Underwater Vision Profiler (UVP) [5], ZOOplankton VISualization system (ZOOVIS) [6], Shadow Image Particle Profiling Evaluation Recorder (SIPPER) [7], and the In Situ Ichthyoplankton Imaging System (ISIIS) [8]. However, *in situ* plankton images are often acquired under sub-optimal conditions, e.g., particulates, turbidity and currents, which can affect light attenuation and scattering and lead to less than ideal image quality [9]. Moreover, imaging systems are capable of generating very large numbers of unique image files [10]. Extracting useful information from a large number of *in situ* plankton images acquired by underwater imaging systems in a timely manner remains a challenge [10, 11]. This latter challenge is essential if *in situ* imaging systems are to achieve their full potential as operational sampling, monitoring, and forecasting tools.

The need for automated plankton recognition and enumeration has spurred the development of new image processing techniques in the past two decades. A typical procedure involves identification and extraction of Regions of Interest (ROIs), feature description, and finally classification into taxonomic categories [10]. For ROI extraction, a common approach is the Otsu global threshold method [12], in which *in situ* images were converted to binary images based on a single threshold value and then connected pixels, i.e., potential targets, were segmented. Another common approach for ROI extraction is the local threshold method represented by the Sauvola’s method [13] in which *in situ* images are converted to binary images using threshold values estimated for different regions. Some techniques also incorporate spatial filtering [14] and color information [15–18] to facilitate ROI extractions. However, for images acquired from highly turbid water, a more complex approach is required. Bi et al. [9] demonstrated that it is more effective to combine Maximally Stable Extremal Regions (MSER) [19] for relatively large targets like jellyfish and the Sauvola’s method to segment small targets like copepods.

Effective feature extraction and description are essential to ensure the success of an automated plankton recognition procedure. Early work often applied feature descriptions based on plankton morphology, for example, a combination of different geometric features [20–22], which appeared to perform well for images acquired under laboratory conditions. Zheng et al. [23] used a basic Local Binary Pattern (LBP) method to describe the texture features of plankton and achieved reasonable results on the microscopic benchmark plankton dataset. Recently many feature descriptors based on local features have been applied in plankton recognition, for example, Histogram of Oriented Gradient (HOG) [9, 23], Scale-Invariant Feature Transform (SIFT) [23, 24] and Shape Context [25].

Classification is the final step in automated plankton recognition in which, each ROI is assigned into one of a number of different classes. Early classification was often based on differential distance measurement, e.g., distance between eigenvectors of feature descriptors [20]. With the recent developments in machine learning, more sophisticated approaches such as Artificial Neural Networks (ANN) [26], random forest classifiers [27], Bayesian approaches [28], and Support Vector Machines (SVM) [9, 29, 30] have been applied to plankton classification. Almost all these existing methods are customized descriptors that achieve the invariance by pre-selected rules and are consequently, not flexible enough to accommodate large variations in image quality and content in plankton images, e.g., morphological variation in the target objects caused by non-uniform illumination. Deep learning methods have been used effectively to provide substantial improvements in image processing and feature extraction and appeared to be good candidates for plankton recognition.

Convolutional Neural Networks (CNN) are a common, deep learning approach, which combines feature description and classification to achieve better performance in various classification tasks. CNN is a weight-sharing network based on image convolution [31]. The convolution result contains a convolution kernel and output. The kernel matches the image features and can be activated for amplification and the output can be used for image classification. The number of CNN models and their capabilities of processing complex images have increased rapidly since the first CNN model (LeNet) was introduced by LeCun et al. [32]. In the past few years, CNNs have moved towards deeper networks to extract complex features and increase accuracy, but increasing network depth often leads to gradient diffusion, which is problematic [32–35]. To overcome the gradient diffusion problem, He et al. [36] introduced a residual block in the neural network, the ResNet. In this new model, convergence speed and identification accuracy were increased by introducing shortcut connections between parameter layers. Another recent development in CNN models is the Dense Convolutional Network (DenseNet) [37], which connects each layer to every other layer in a feed-forward fashion. This alleviates gradient loss and reuses features learned before as depth increases. The disadvantage is that DenseNet implementation can require a large amount of memory.

The application of CNNs to plankton recognition and enumeration is still relatively new. Ouyang et al. [38] implemented a multi-size image sensing module and a deep CNN to identify 121 plankton species from 2015 National Data Science Bowl. Li et al. [39] employed deep ResNet to identify these 121 types of plankton, too. However, both studies used images acquired under ideal imaging conditions. In contrast, processing *in situ* plankton images is more challenging. Luo et al. [40] applied a sparse CNN model to process images acquired from the In Situ Ichthyoplankton Imaging System (ISIIS). The advantage of a sparse CNN is that each sparse convolutional layer can be performed with a few convolution kernels followed by a sparse matrix multiplication, which leads to higher computation efficiency [41].

The effectiveness of a CNN model for plankton recognition could affected by the presence of substantial noise due to less than ideal imaging conditions, and ambiguous features in the descriptors, boundaries, and internal features. Meanwhile, low plankton abundances, i.e., low number of occupied pixels by potential target objects would clearly exacerbate the feature problem. For example, an *in situ* image from the ZOOplankton VISualization (ZOOVIS) system has dimensions of 2448×2044 pixels and even large planktonic organisms such as small jellyfish have average pixel dimensions < 200×200 [42]. After multiple layers of convolution and pooling, the detailed features will continuously disappear, resulting in a gradual reduction in the number of valid features retained. Lastly, the diversity of plankton in the oceans is high and many plankton species share similar morphological features. The lack of clearly defined features could be exacerbated by the less than ideal imaging conditions which makes it difficult to apply a model with a deep network structure. CNNs have an unrivaled feature description capability, and the key for an successful implementation of a CNN for *in situ* plankton images is to reduce the impact of unambiguous features at the input end of CNNs, i.e., improve morphological feature for better feature description, and adopt a more plankton-targeted classifier at the output end of CNNs.

In this paper, we implemented an end-to-end *in situ* plankton image identification and enumeration method. To reduce the impact of unambiguous features, we developed a specialized adaptive ROI extraction and feature enhancement procedure. For better classification, we built a multi-class SVM model to achieve global optimization of the learned feature among different target groups. To identify the proper CNN model for plankton recognition, we compared the performance of several readily available CNNs on the same plankton dataset including AlexNet [31], VGGNets [43], GoogleNet [44], and ResNet. AlexNet model [31] was proposed in 2012, which contains 5 convolution layers and 3 fully connected layers, and this model used big data, Graphics Processing Unit (GPU), Rectified Linear Units (ReLU), and dropout techniques to accelerate network convergence speed while simultaneously preventing overfitting. VGGNets [43] have fewer parameters by performing multiple continuous small-scale convolutions instead of one step of large-scale convolution to gain more non-linear expressions and achieve a better performance with less parameters. GoogLeNet model [44] used the inception structure to replace simple traditional operations of convolution and activation to better tackle the large variation in ROIs to achieve better performance. The core idea of this model is to use wide inception structure to make models automatically adapt to features at different scales. ResNet implemented shortcut connections and showed advantages in convergence speed and identification accuracy [35, 36]. The introduction of shortcut guarantees that the model makes full use of network residual information, which makes the topology of the network more complex, and improves the performance of the model with a much deeper layers.

## Methods

### Data description

**Fig 1:**
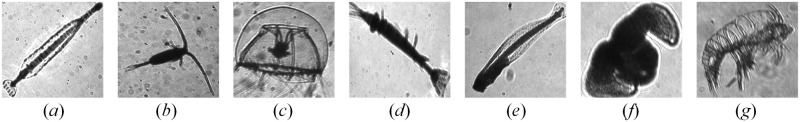
Example images of 7 classes in our *in situ* plankton dataset. (*a*) chaetognatha; (*b*)copepoda; (*c*) medusae; (*d*) euphausiids; (*e*) fish larvae; (*f*) Limacina; (*g*) other zooplankton or objects.

The plankton images used in this study were obtained by the ZOOVIS underwater *in situ* imaging system that was deployed in the southeastern Bering Sea in May 2017. The datasets used for training and testing were obtained from these *in situ* images (685,520 in total) using the ROI extraction procedure described below. The segmented images were manually separated into different taxa and verified by one of the authors (HB). The sample datasets (Fig 1) used in the experiments included 6 plankton categories (chaetognatha, copepoda, medusae, euphausiids, fish larvae, and limacina) and an ‘other’ category to accommodate zooplankton other than the six primary categories and non-zooplankton particles. Constructing and training CNN models requires tens of thousands of images which is problematic for rare plankton classes. For example, there were insufficient chaetognatha, medusae, euphausiids and fish larvae present in the data to establish a training set with an adequate number of images. Under these circumstances, we rotated and mirrored existing ROIs and artificially created ROIs to mimic real ROIs. For example, the back view of a copepods could be rotated to represent different orientations. The treatment details for rare classes varied slightly due to difference in their occurrence (euphausiids > chaetognatha and medusae > fish larvae). Each euphausiid ROI was rotated by 90 degree and 180 degree clockwise, and mirrored from up to down, which led to a threefold increase in sample size. Each chaetognatha and medusae ROI of them was rotated by 90 degree and 180 degree, and mirrored from up to down and left to right, which leads to a fourfold increase in sample size. The number of fish larvae ROI had the least occurance, so in addition to the rotation by 90 degree and 180 degree, and mirror from up to down and from left to right, the contrast of each ROI of this category is adjusted with a wider dynamic range of grayscale so as to mimic different imaging environment, which led to a fivefold increase in sample size. All this sample expansion methods are best chosen to mimic the real underwater *in situ* imaging environment. Using this approach we were able to ensure that there were 2048 ROIs in the training set and 512 ROIs in the testing set for each class and therefore we could train a balanced and unbiased classifier. To ensure an independent test, all the samples used in the training set did not appear in the testing set. The expanded training data set and testing test are to take into account various motion patterns, different oritentations and various underwater imaging environment. A more inclusive sample library could mimic plankton details in real water bodies and allow more accurate and realistic plankton feature description. Finally, we adjusted the contrast level (*δ*) to mimic different imaging conditions. We chose 5 contrast levels: 3.1, 3.3, 3.5, 3.7, and 3.9 based on imaging conditions presented in the dataset. At this point, the number of samples in the training set was 10,240 for each class. The dataset of in situ plankton images is available online: DOI: 10.6084/m9.figshare.8146283.

### Description of the procedure

**Fig 2:**
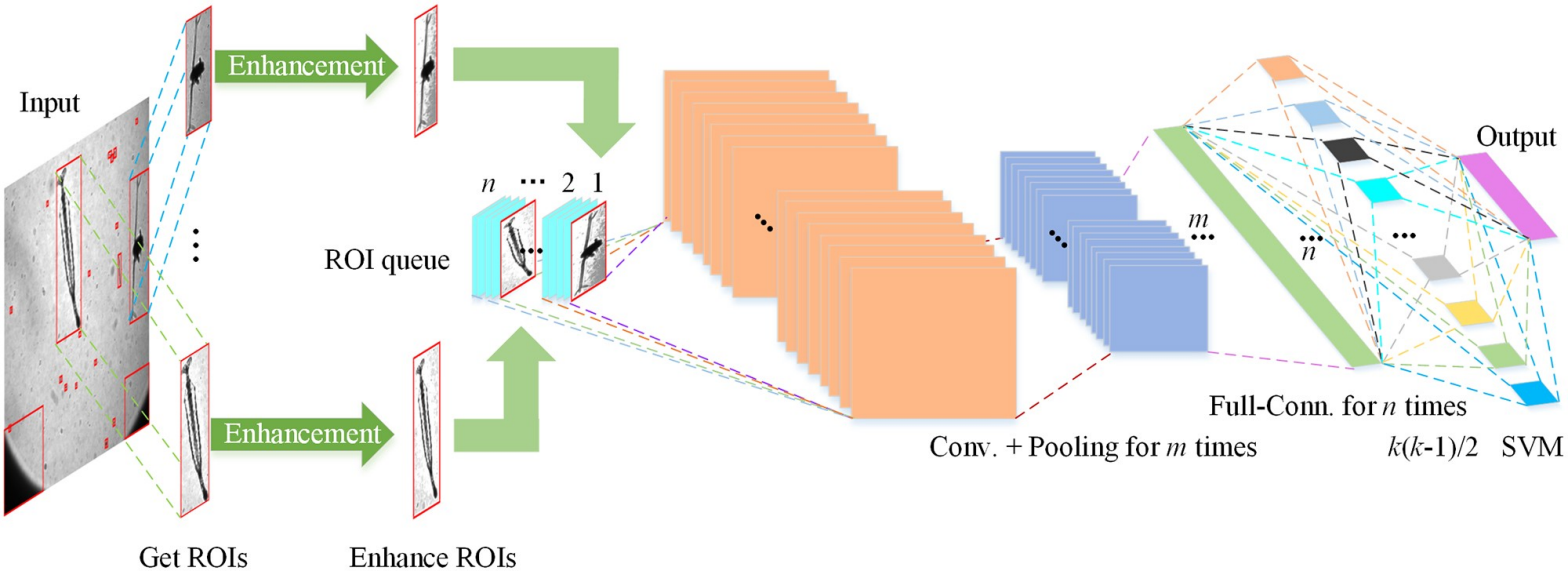
A flow chart illustrating the different steps and modules in the proposed automated plankton identification and enumeration procedure.

Image convolution is a key process in CNNs in which image features are activated, amplified, and outputted under suitable convolution kernel parameters. The effectiveness of features of the image will directly affect the final output. To overcome the problems of *in situ* plankton images discussed previously, the proposed procedure started from designing an algorithm that can effectively extract potential target objects from images with highly variable contents and different levels of edge sharpness and object contrast ratio (Fig 2). The potential target objects (ROIs), were first located and segmented. Subsequently, local grayscale values were used to enhance the local features of ROIs to allow more effective feature description and reduce gradient loss during the convolution process. Through the image convolution, the features of the enhanced ROIs were extracted after the fully connected layer was achieved in the network. The extracted feature was used as the input for the SVM model classification. In summary, the procedure includes 4 modules: adaptive ROI extraction, ROI enhancement, feature extraction by the CNN, and the multi-class SVM model.

### Adaptive ROI extraction

**Fig 3:**
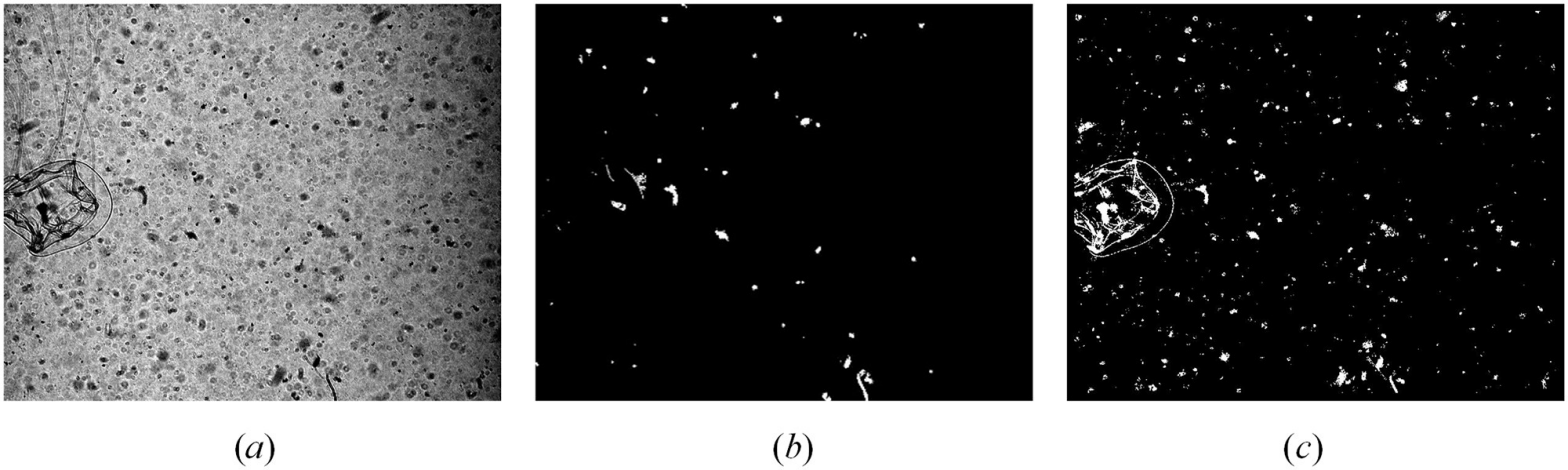
Examples of binarization with global threshold and local threshold methods. (*a*) Original image with uneven illumination, the contrast of which is adjusted only for illustration purpose due to the heavy darkness of original image, and the procedure directly use the original image; (*b*) Result from binarization with global threshold method; (*c*) Result from binarization with Sauvola’s method based on sliding window.

The objective of ROI extraction is to separate the target object from a complex and noisy background and to reduce the interference of the background noise and improve the feature extraction in the subsequent image convolution within the CNNs (Fig 3(*a*)). Low levels of edge sharpness and ambiguous morphological features make it difficult to extract ROIs. For example, the commonly used Otsu’s global threshold method missed many target objects (Fig 3(*b*)). A combination of Maximally Stable Extremal Regions (MSER) [19] and Sauvola’s local binarization method [13] appeared to a good choice [9]. A caveat is that both methods need to manually specify parameters to extract ROIs. MSER requires manual specification of the maximum area variation between extremal regions and step size between intensity threshold levels. Sauvola’s local binarization requires a manual specification of suitable window size and a fixed coefficient *k* in Sauvola’s method (Eq 3) to obtain intact target regions. Another problem is that both MSER and Sauvola’s method have trouble in segmenting targets with multi-parts and different levels of edge intensity. Uneven illumination and varying contrast ratio within the same image can lead to excessive ROIs for both methods.

To overcome these issues, we described the image contrast ratio using Mean Signal-to-Noise Ratio (*MSNR*), which is defined by Eqs 1 and 2.

$$M=\frac{\left(x_{1}+x_{2}+\ldots +x_{n}\right)}{n},$$ (1)

$$MSNR=\text{max}{\left(M-x_{i}\right)}^{2},i=1,2,\ldots ,n,$$ (2)

where *n* represents the number of pixels, *M* represents the mean value of pixels in the entire image, *x*_*i*_ represents the *i-*th pixel, and max is the maximum pixel value. For *in situ* plankton images, when the *MSNR* is small, the changes in the contrast ratio and the sharpness of the whole image are low, and it is not easy to distinguish target objects from the background and particulates. When the *MNSR* is large, the whole image is clear, and it is relatively easy to extract target objects.

In the present study, we used a threshold value of 0.1 for *MSNR*. For images with a high contrast ratio, *MSNR*>0.1, we used the MSER method to segment these images. For images with a low contrast ratio, *MSNR*≤0.1, which is often the case for *in situ* plankton images, we used the Sauvola’s method, a local threshold segmentation approach to extract ROIs. First, each pixel was considered as a center, and a sliding window was used for pixel-by-pixel sliding on the image with a step of 1 pixel. The length and width of the sliding window was 1~3% of the entire image size. Within every sliding window, we first employed the Sauvola’s method to obtain the local threshold value within the sliding window (Eq 3).

$$T(x,y)=m(x,y)[1+k(\frac{\delta (x,y)}{R}-1)],$$ (3)

where *T*(*x*, *y*) represents the threshold value for the sliding window at (*x*, *y*) calculated from the local contrast ratio, *R* represents the maximum standard deviation of all pixels in the image that may occur which is 128 for a grayscale image [13], *k* is a fixed coefficient which usually has a value of 0.34, *m*(*x*, *y*) is the mean pixel value of all pixels in the sliding window and *δ*(*x*, *y*) is the standard deviation of all pixels in the sliding window. The necessary source codes in MATLAB language for ROI extraction are available here: https://github.com/KaichangCHENG/PIE-MC/tree/master/EnhancedCNN.

The standard Sauvola’s method only binarizes the central pixel during each sliding and the sliding step length is 1 pixel and each window has overlapping positions. Based on the analysis, when the changes in pixel values within the region are large, the contrast ratio is large, and the regional standard deviation *δ*(*x*, *y*) will approach the maximum standard deviation *R*, i.e., *T*(*x*, *y*) ≈ *m*(*x*, *y*). However, in regions with a lower contrast ratio, *T*(*x*, *y*) is significantly lower than *m*(*x*, *y*). The single-pixel sliding Sauvola’s method allows an adaptive threshold value, ensuring that the threshold value is between the potential target object and background noise and distinguish them effectively. For example, we applied this technique on the same *in situ* image for Otsu’s global thresholding approach and the single-pixel Sauvola’s method performed much better in which the ROIs were separated and extracted from the background effectively (Fig 3(*c*)).

### ROI enhancement

After extracting the ROIs for the potential target objects, we implemented a procedure to enhance the morphological features of ROIs and suppress background noise.

#### Target feature enhancement

**Fig 4:**
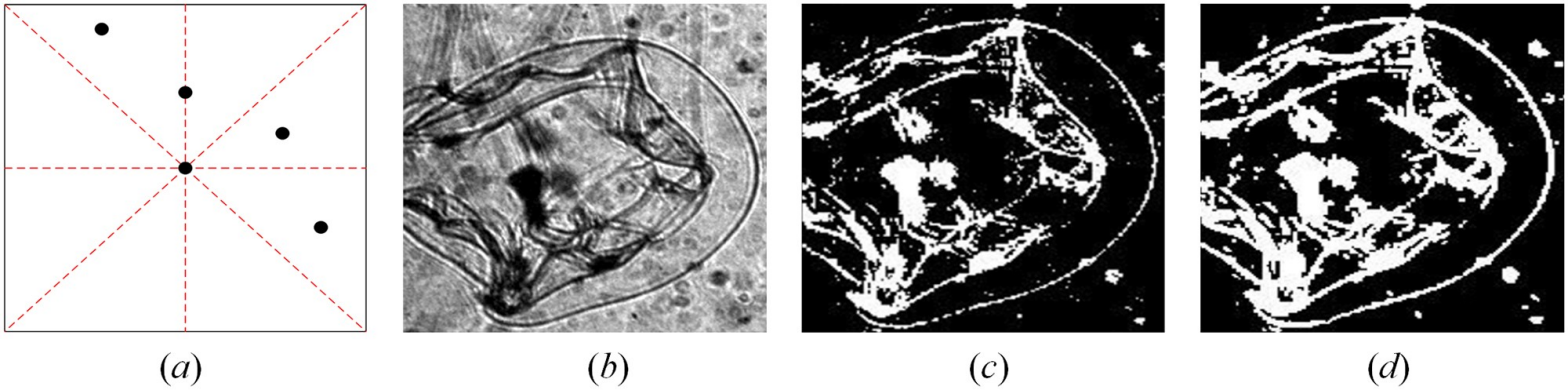
Enhancing target features. (*a*) Illustration of valid pixel. The black point in the center of the black rectangle is the pixel to be confirmed, and the 0.75*N*_*valid*_ valid pixels (other black points) around it lie within one of the rectangular windows or between two adjacent rectangular windows, so the central pixel is a part of the target; (*b*) Original ROI, the contrast of which is adjusted only for illustration purpose due to the heavy darkness of original ROI, and the procedure directly use the original ROI; (*c*)Example from binarization with Sauvola’s method; (*d*) Example after enhancement with the edge roughening method.

Due to the complexity of plankton images, conventional spatial domain filtering and frequency domain filtering were not effective. The main inherent problem was too many breakpoints in the target region, making it difficult to extract intact targets. To solve this problem, we employed a denoising algorithm based on breakpoint connections in the spatial domain to achieve target feature enhancement. Based on using the above method for ROI extraction, we took every pixel as a center and use a small rectangular window that was 1~3% of the complete ROI to segment it into many small units. Subsequently, Eq 4 was used to calculate threshold values for every small unit. White pixels were considered as valid pixels and the number of valid pixels within this rectangular region (labeled as *N*_*valid*_) was compared with the threshold value to determine whether this central pixel is a biological boundary feature point:

$$T_{value}=floor(\sqrt{2{\left[floor(\sqrt{N_{rect}})\right]}^{2}})-2,$$ (4)

where *T*_*value*_ represents the adaptive threshold value, *N*_*rect*_ is the number of all pixels in the rectangular window that we set, and the *floor* equation represents a rounding down. The valid pixels in 0.75*N*_*valid*_ had to lie within one of the rectangular windows or between two adjacent rectangular windows, otherwise that region was regarded as background or noise (Fig 4(*a*)).

The reasons for these settings are twofold. When a valid pixel is the boundary or internal feature point of plankton, there will be many identical feature points nearby. Therefore, the number of valid pixels will be greater than the length of the diagonal line of the rectangular window. From the perspective of pixel density, this can be interpreted as a form of expression for high frequency information. Secondly, if that pixel is a feature point, then its surrounding pixels will be distributed around it according to specific rules and will not be scattered randomly within the rectangular window. Therefore, we can carry out filter denoising of the image from the angle of the spatial domain. Fig 4(*c*) and 4(*d*) shows the raw extracted ROI using the sliding window described in the previous section and the results after thickening of the target boundaries through determination of valid pixels, respectively.

#### Background suppression

After obtaining the intact plankton feature image, the pixel intensity for the segmented ROI was stored in an array **P**_*back*_ and the locations of target object in array **P**_*back*_ were all set to 0. The remaining nonzero pixel intensity in **P**_*back*_, denoted as *p*, were arranged according to the pixel values, and the boundary threshold *T*_*b*_ was set (Eq 5).

$$T_{b}=p_{\text{min}}+\frac{p_{\text{max}}-p_{\text{min}}}{\delta },\delta \in [3,4],$$ (5)

where *p*_min_ represents the lowest pixel value in the region, *p*_max_ represents the largest pixel intensity value in the region, and *δ* is the boundary parameter, which is usually a number between 3 and 4.

**Fig 5:**
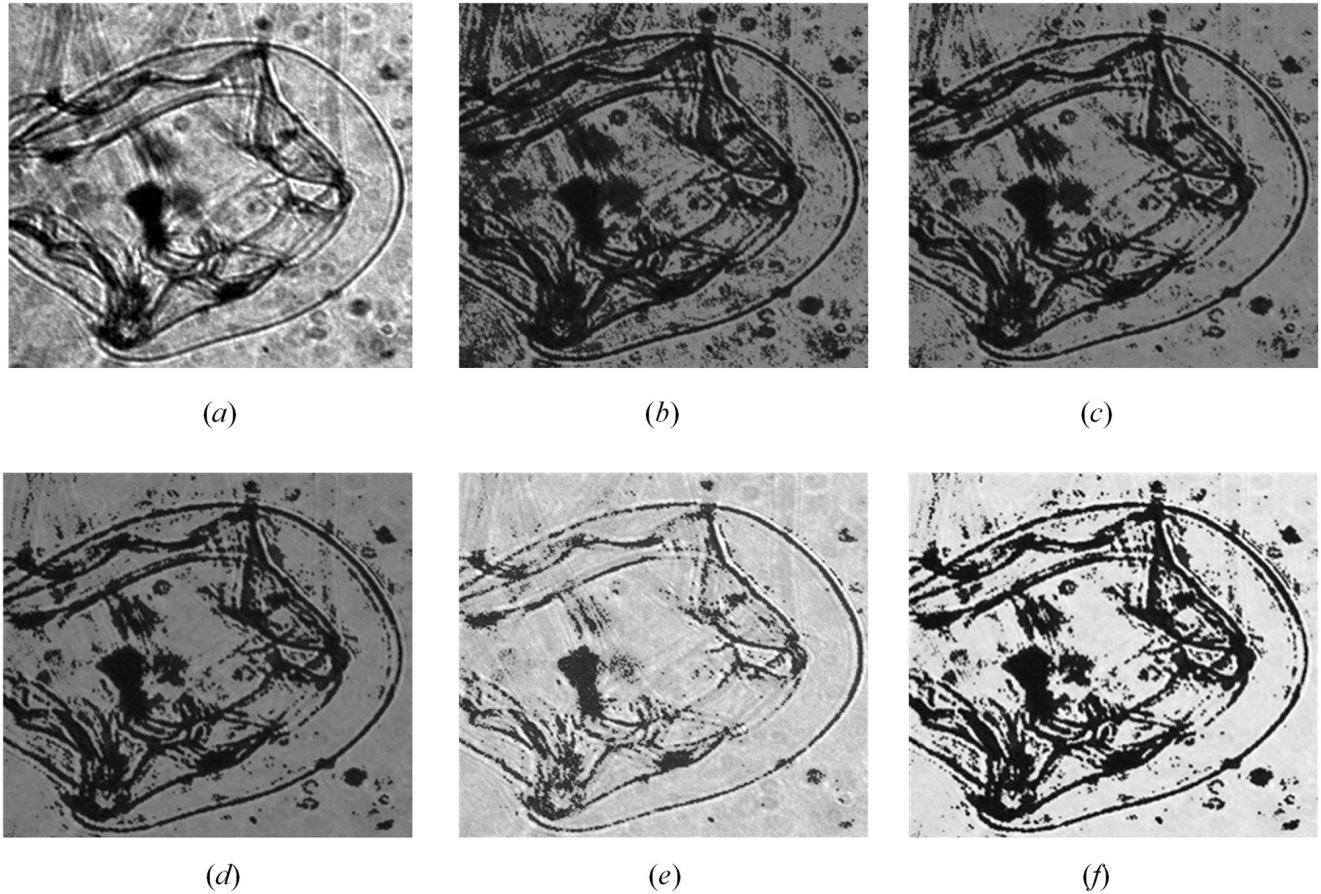
Effects of different *δ* on ROI enhancement. (*a*) Original ROI, the contrast of which is adjusted only for illustration purpose due to the heavy darkness of original ROI, and the procedure directly use the original ROI; (*b*) *δ* = 3.1; (*c*) *δ* = 3.3; (*d*) *δ* = 3.5; (*e*) *δ* = 3.7; (*f*) *δ* = 3.9.

**Fig 6:**
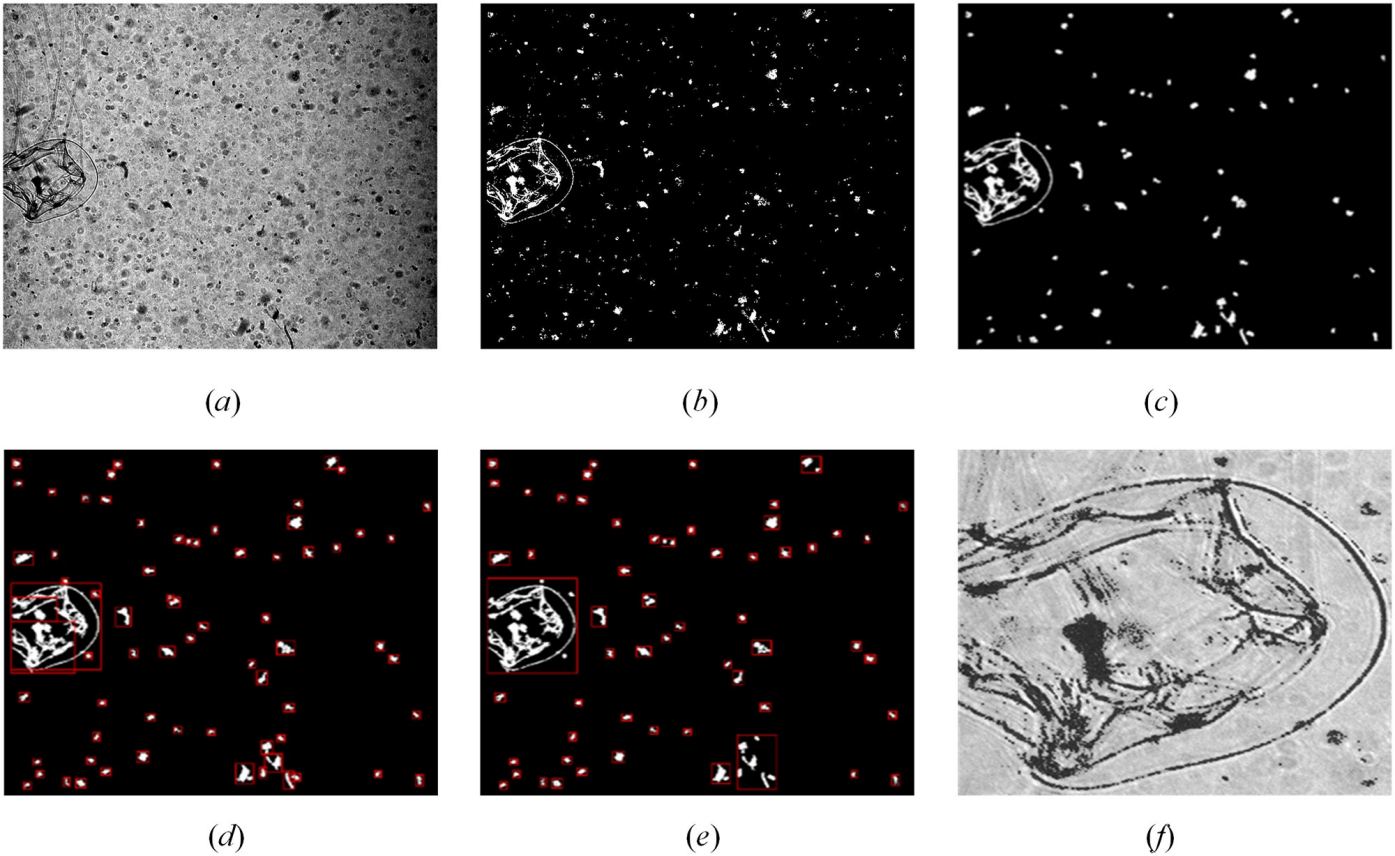
Effects of ROI enhancement with different steps. (*a*) Original plankton image, the contrast of which is adjusted only for illustration purpose due to the heavy darkness of original image, and the procedure directly use the original image; (*b*) The effect of binarization with Sauvola’s method; (*c*) The effect of denoising and edge roughening; (*d*) Extraction of ROIs based on connected domain; (*e*) Extraction of ROIs with rectangular merging method based on RPN [45]; (*f*) Final enhanced ROI after background suppression, *δ* = 3.7.

Grayscale transformation *p*′ = *p* + 5 ·(*p* − *T*_*b*_)·(*δ* − 3) was carried out based on the difference between the pixel value and the threshold value *T*_*b*_ to get new background intensity *p*′, where *p* is the nonzero pixel values in **P**_*back*_. Pixel values lower than the threshold value were suppressed and pixel values greater than threshold value were artificially amplified due to the changes in relative intensity. Through this approach, the differences between different pixels were enhanced and morphological features were better reflected. Finally, the new transformed pixel intensity p′ were used to replace *p* that were recorded in array **P**_*back*_. Note that changes in boundary parameter affect morphological features. For example, when the boundary parameter ranged from 3.1 to 3.9, the morphological features of potential object were enhanced substantially (Fig 5). In the present study, we set the boundary parameter to 3.7 to achieve an optimum result for ROI enhancement (Fig 6). The necessary source codes in MATLAB language for ROI enhancement are also available here: https://github.com/KaichangCHENG/PIE-MC/tree/master/EnhancedCNN.

### CNN models for feature learning

CNNs are commonly used in pattern recognition with superior feature learning capabilities. When applied to plankton recognition, it is important to determine the best suitable network structure to overcome issues in plankton recognitions. For example, many plankton are small with mesozooplankton ranging from 200 μm to 2,000 μm and microzooplankton ranging from 20 μm to 200 μm. They can have a wide range of morphological features and sometimes it is difficult to distinguish them from non-living particulates in the water column. In the present study, we tested common network structures for plankton recognition including AlexNet, VGG16, VGG19, GoogLeNet, and ResNet and compared their performance. To examine the impact of ROI enhancement at the front end of the model and the multi-class SVM model at the back of the model, we directly used the convolutional and fully connected layers in CNN models for the feature learning and we used samples in our training set to fine-tune the CNNs. In the back end of the classification model, we used the output of the fully connected layer in these classical CNNs as extracted ROI features, the input for the multi-class SVM model training and classification.

### Multi-class SVM classification

**Fig 7:**
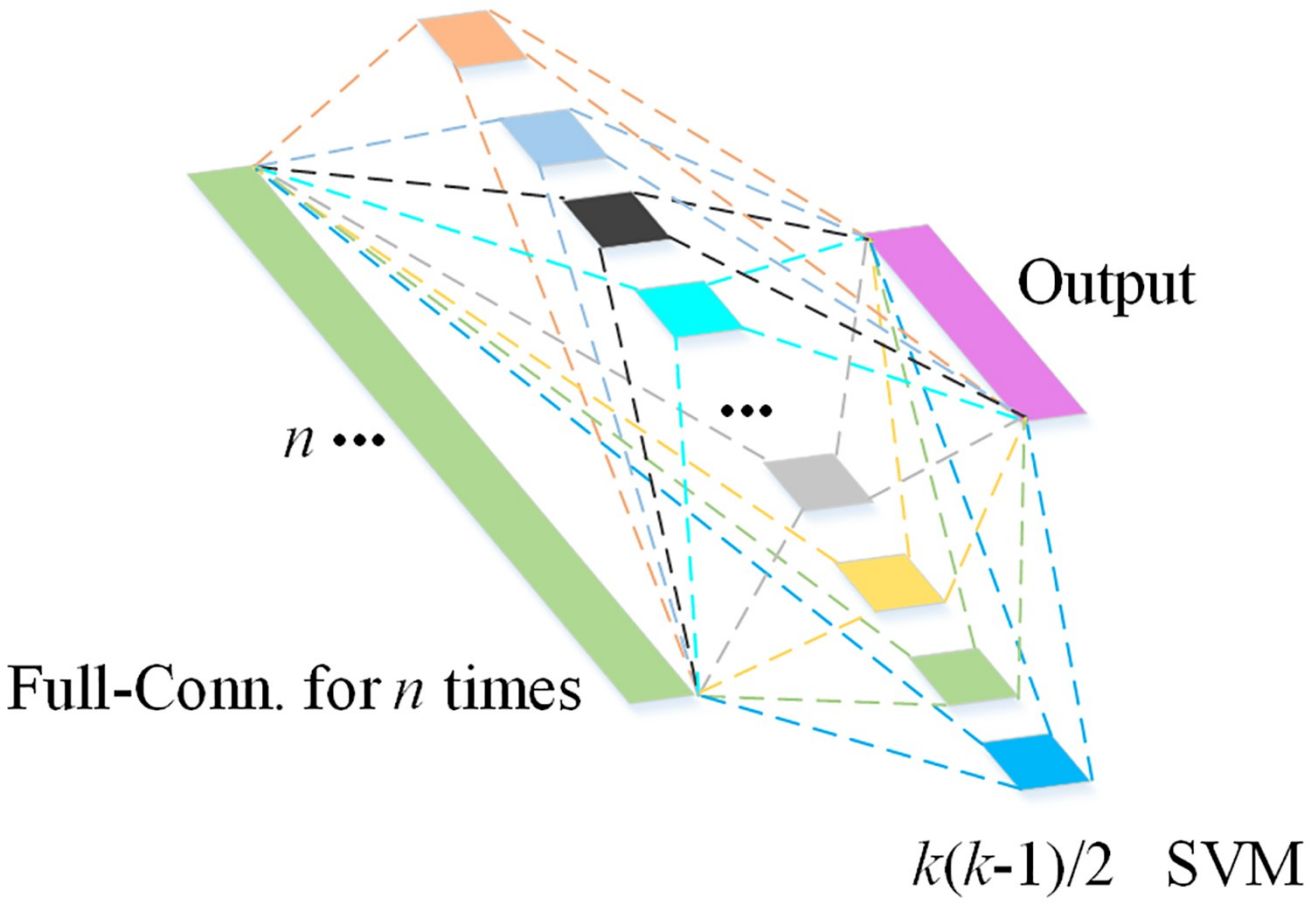
Illustration the multi-class SVM model starting from pairwise classification to the final output.

The advantage of SVM is that it uses support vectors to identify optimal hyperplanes in a feature space so that the distance between positive and negative samples in the training set is maximized. In addition, flexible intervals can be used to increase fault tolerance and improve the robustness and classification accuracy. Regarding plankton recognition, species within the same class may show large variation, e.g., different copepod species vary in size and morphological features, and therefore a model with high fault tolerance would be beneficial for classification. In this paper, we employed a multi-class SVM model (Fig 7) to classify target objects using features extracted from selected CNNs.

In the proposed multi-class SVM, a one vs. one classification will train 1 classifier between every two classes. Therefore, there will be *k*(*k* − 1)/2 classifier functions for a problem with *k* classes. We used a simple linear classifier, *f*(**X**) = **W**^T^**X** + *b* to map each ROI to different classes, where **W** is a weight vector, **X** is a feature vector, and *b* is the bias. When the trained model is used for classification of unknown samples, every classification function will be used to determine its class and the probability of its class. The class of the unknown sample corresponds to the class with the highest probability. As a multi-class SVM model will carry out a 1 vs. 1 comparison between every two classes, it will increase computational demands for identification but effectively increase classification accuracy as compared with a one vs. all SVM model. There were 7 different groups of plankton in the present study groups. Therefore, the computational demand for the 1 vs. 1 multi-class SVM in this study was not increased too much. More importantly, the structural diversity of non-target objects was extremely high, which required a classification model with high accuracy.

## Results and discussion

To validate the accuracy and efficiency of the enhanced CNN proposed in this study, we conducted multi-group comparison and validation experiments. The experiments were conducted using MATLAB (Release 2018a). The accuracy, recall, and elapsed time were the mean values of 5 replicates.

The first experiment (Table 1) was designed to examine whether incorporate the multi-class SVM would improve the performance of the CNN models. The fully connected layers from the selected CNN models were used to describe sample features and were used as the input for the multi-class SVM model. For CNNs with fully connected layers (AlexNet, GoogLeNet, VGG16 and VGG19), the output of corresponding fully connected layers is a one-dimensional feature vector, which was used as the input of multi-class SVM model. While there is no real fully connected layer in ResNet50 model, the output of the last dropout layer in this model is a one-dimensional feature vector, which could be used as the input of multi-class SVM model. The baseline based on Histogram of Oriented Gradient (HOG) features and SVM classifier had relatively low performance on *in situ* plankton ROIs with precision and recall rates of approximately 60%. The selected classic CNN models without the multi-class SVM performed much better on the same set *in situ* plankton training set with both precision and recall rates ranging 85% ~ 88%. Within this group, the ResNet50 model performed the best. Model128_5 and model48_5 proposed by Ouyang py et al. [38] designed for the classification of 2015 National Data Science Bowl with a good imaging quality and did not perform well for *in situ* plankton images, which is only 71.26% and 67.16%, respectively. When the selected CNN models were combined with the multi-class SVM, i.e., some fully connected layers and Softmax classification layer in classic CNN models were replaced by the multi-class SVM model, both the precision and recall rates increased to 88% ~ 92%. We did not use the last fully connected layer of each classic CNN model because it was a special vector that included the corresponding scores of all the classes to be predicted in the model, not the learned features. From Table 1, we can see that the classification performance of experiment using specified features learned from rear fully connected layers were not as good compared with those that directly used the first layer, except for the AlexNet Model. Therefore, we used the output from the fully connected layer of the classical CNN, which was proved to have a better performance in Models 7 to 14, as the input for the multi-class SVM model. The necessary pre-trained models in MATLAB language are also available from https://doi.org/10.6084/m9.figshare.8146283.

**Table 1:** Results of model performance.

Treatments	Model (Detailed structure)	Precision (%)	Recall (%)	Time (ms/sample)
Baseline	HOG + Multi-class SVM	61.33	60.24	16.82
CNN models without SVM	AlexNet^a^	85.55	85.06	25.95
	GoogLeNet^b^ [38]	86.87	87.01	84.10
	VGG16^b^ [38]	86.18	86.87	341.59
	VGG19^b^ [39]	87.03	87.11	410.76
	ResNet50^b^ [39]	88.22	88.46	172.26
	model128_5^b^ [38]	71.26	71.87	186.32
	model48_5^b^ [38]	67.16	67.89	92.45
CNN models with SVM	AlexNet-fc1 + Multi-class SVM	88.63	88.15	29.05
	AlexNet-fc2 + Multi-class SVM	89.01	88.62	33.09
	GoogLeNet-fc´ + Multi-class SVM	90.45	90.15	87.70
	VGG16-fc1 + Multi-class SVM	91.33	91.13	371.27
	VGG16-fc2 + Multi-class SVM	90.18	90.75	375.62
	VGG19-fc1 + Multi-class SVM	91.86	91.42	425.72
	VGG19-fc2 + Multi-class SVM	90.88	90.99	428.81
	ResNet50-fc´ + Multi-class SVM	92.47	92.76	176.76

**Note:** Symbol fc1 and fc2 indicate that learned features are from the corresponding fully connected layers according to the order in CNN models. Some models only have one fully connected layer, and outputs of this fully connected layer are the corresponding scores of all the classes to be predicted in the model, not learned features. For GoogLeNet and ResNet50, we use fc´ to indicate that the learned features are from the last dropout layer in the CNN, the output of which is a vector and similar to the output features of fully connected layer in other CNN models. HOG indicates histogram of gradients as feature descriptor and SVM represents support vector machine for classification.

^a^No one used this model to identify plankton images before, and the result reproduced based on *in situ* plankton dataset made by our own group is only for reference.

^b^These models were used for the classification of 2015 National Data Science Bowl with a good imaging quality firstly, and the results in the table were reproduced based on *in situ* plankton dataset made by our own group.

**Fig 8:**
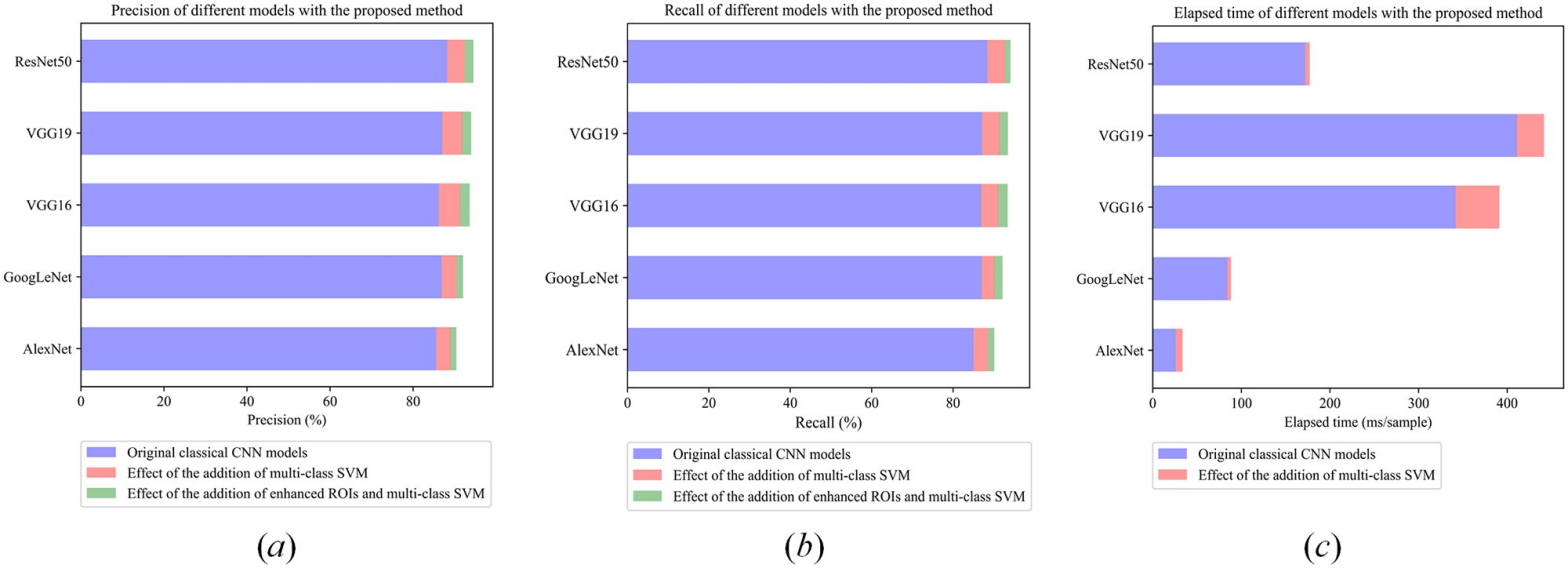
Impacts of multi-class SVM and enhanced ROIs. (*a*) Precision of different models with the proposed method; (*b*) Recall of different models with the proposed method; (*c*) Elapsed time of different models, the increasement of elapsed time (in light coral) is the average of corresponding models based on original ROIs and enhanced ROIs.

Because the original ROIs contained a lot of noise, the second experiment (Table 2) was performed to examine the impact of ROI enhancement on the precision and recall rates using the same selected CNNs, feature output and the SVM classifier. We used different *δ* values to enhance the original ROI, and then the original samples and all the enhanced samples were combined together to train the classifier. Note that the spatial domain-based breakpoint connection ROI enhancement method was only applied on samples in the training set, while samples in testing set used for performance test were not enhanced. The classification precision, recall, and time consumed generally increased by 1~2% (Fig 8).

**Table 2:** Effect of ROI enhancement in plankton identification and enumeration.

No.	Model (Detailed structure)	Precision (%)	Recall (%)	Time (ms/sample)
1	OriginalROI + AlexNet-fc2 + Multi-class SVM	89.01	88.62	33.09
2	OriginalROI + GoogLeNet-fc´ + Multi-class SVM	90.45	90.15	87.70
3	OriginalROI + VGG16-fc1 + Multi-class SVM	91.33	91.13	371.27
4	OriginalROI + VGG19-fc1 + Multi-class SVM	91.86	91.42	425.72
5	OriginalROI + ResNet50-fc´ + Multi-class SVM	92.47	92.76	175.76
6	EnhancedROI + AlexNet-fc2 + Multi-class SVM	90.44	90.13	34.13
7	EnhancedROI + GoogLeNet-fc´ + Multi-class SVM	92.04	92.15	88.90
8	Enhanced ROI + VGG16-fc1 + Multi-class SVM	93.65	93.43	411.25
9	EnhancedROI + VGG19-fc1 + Multi-class SVM	93.99	93.48	427.57
10	EnhancedROI + ResNet50-fc´ + Multi-class SVM	94.52	94.13	178.42

**Fig 9:**
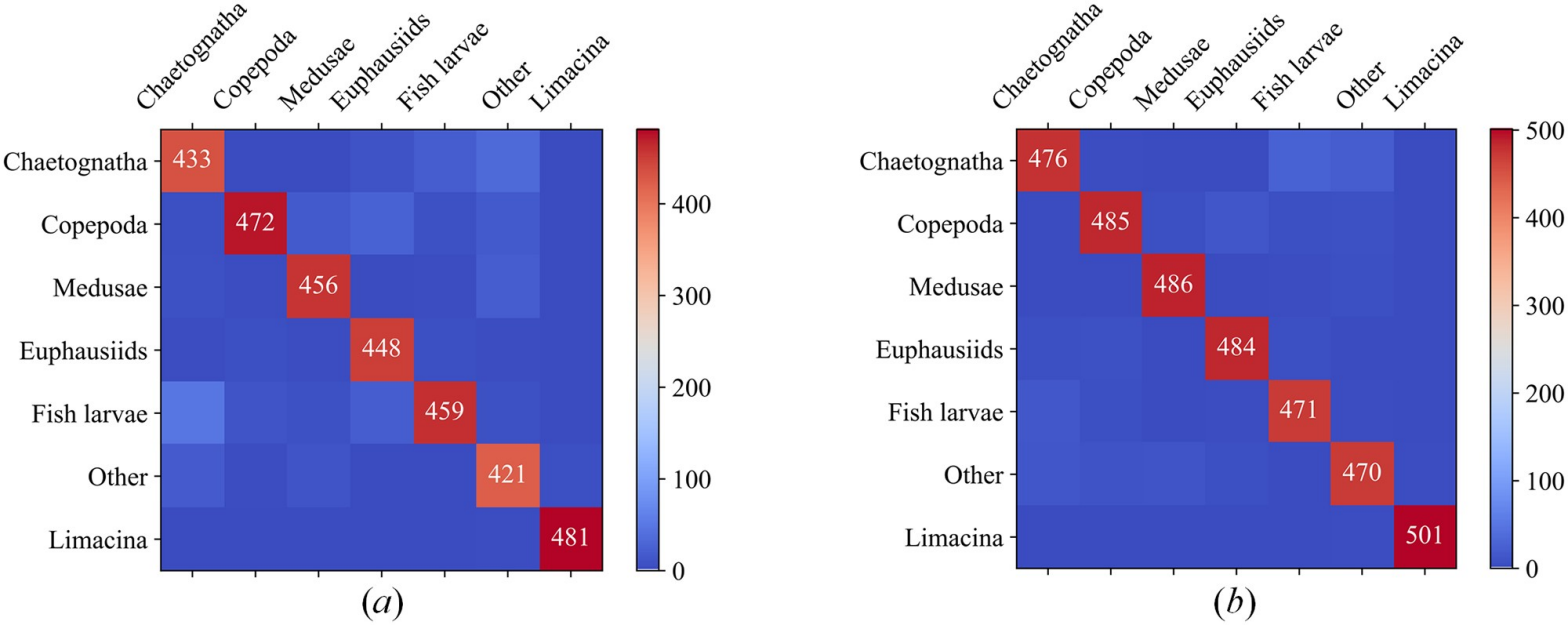
Confusion matrix of the final results of original ResNet50 and the selected optimal-performance model. (*a*) Confusion matrix of original finetuned ResNet50; (*b*) Confusion matrix of EnhancedROI + ResNet50-fc´ + Multi-class SVM.

When compared to the original classical CNN model, the combination of feature enhancement and multi-class SVM classification layers can increase the classification accuracy and recall of the model by 3~6%, while the computing time increased only by 7~10%. Results suggested that ResNet50 combined with the multi-class SVM performed the best with precision and recall rates >94% and average processing time ~176.764 ms/sample. The misclassification rates among the selected 7 classes also declined using the proposed procedure, i.e., EnhancedROI + ResNet50-fc´ + Multi-class SVM when compared to the results using ResNet50 model alone. Fig 9 showed the corresponding confusion matrix of 7 classes for original finetuned ResNet50 Model and the optimal-performance model proposed in this paper, namely, EnhancedROI + ResNet50-fc’ + Multi-class SVM. Clearly, the latter had a much better performance. From the confusion matrix, we can see that limacine is the easiest to identify due to its simple and fixed shape. Copepoda, medusae and euphausiids are relatively harder to identify for their various motion patterns. Chaetognatha, fish larvae and other category are the hardest to identify because of their flexible shapes and motion patterns. Detailly, chaetognatha and fish larvae always have a long and thin body, behaviors of which are always similar and indistinguishable, so the identification results were relatively poor. Other category contains zooplankton other than the six primary categories and non-zooplankton particles which have various behaviors and shapes, so the identification performance is the worst of the 7 classes.

## Conclusions and prospects

In summary, we examined the effectiveness of different CNNs models in describing plankton features and results suggested that the ResNet50 performed the best among the 6 selected CNN models. The advantage of ResNet50 in describing plankton likely rises from its relatively wide network structure which allows a better description of plankton, often with relatively small size and < 100 pixels. The inclusion of a multi-class SVM classification model improved the robustness and classification accuracy of the proposed procedure. Finally, a dedicated ROI enhancement helped to remove the background nose and allows more effective feature description which subsequently improved the performance of the proposed procedure with both precision and recall rates >94%. We concluded that the selected ResNet50 model structure combined with the ROI enhancement and the multi-class SVM classification model could effectively identify and enumerate plankton for optical plankton imaging systems like ZOOVIS and other *in situ* plankton imaging systems.
